# N-Homocysteinylation Induces Different Structural and Functional Consequences on Acidic and Basic Proteins

**DOI:** 10.1371/journal.pone.0116386

**Published:** 2014-12-31

**Authors:** Gurumayum Suraj Sharma, Tarun Kumar, Laishram Rajendrakumar Singh

**Affiliations:** Dr. B. R. Ambedkar Center for Biomedical Research, University of Delhi, Delhi, India; CSIR-Institute of Genomics and Integrative Biology, India

## Abstract

One of the proposed mechanisms of homocysteine toxicity in human is the modification of proteins by the metabolite of Hcy, homocysteine thilolactone (HTL). Incubation of proteins with HTL has earlier been shown to form covalent adducts with *ε*-amino group of lysine residues of protein (called N-homocysteinylation). It has been believed that protein N-homocysteinylation is the pathological hallmark of cardiovascular and neurodegenerative disorders as homocysteinylation induces structural and functional alterations in proteins. In the present study, reactivity of HTL towards proteins with different physico-chemical properties and hence their structural and functional alterations were studied using different spectroscopic approaches. We found that N-homocysteinylation has opposite consequences on acidic and basic proteins suggesting that pI of the protein determines the extent of homocysteinylation, and the structural and functional consequences due to homocysteinylation. Mechanistically, pI of protein determines the extent of N-homocysteinylation and the associated structural and functional alterations. The study suggests the role of HTL primarily targeting acidic proteins in eliciting its toxicity that could yield mechanistic insights for the associated neurodegeneration.

## Introduction

Hyperhomocysteinemia/homocystinuria is a genetic disorder of methionine metabolism caused due to elevated level of plasma homocysteine (Hcy). The toxic Hcy is known to metabolize to methionine by remethylation or to cystiene by trans-sulfurylation. However, mutations in the Hcy metabolizing enzymes, cystathionine β-synthase (CBS) or methylene tetrahydrofolate reductase (MTHFR) cause an impaired ability to metabolize the toxic Hcy resulting in an increased levels of cellular and plasma Hcy [1], [2], [3], [4], [5]. The total concentrations of plasma Hcy may range from 15–20 µM (mild forms) up to 500 µM (severe forms), compared with 5–10 µM under normal conditions [6], [7], [8], [9], [10], [11]. This elevated Hcy levels are associated with increased incidences of cardiovascular diseases, including atherosclerosis and thrombosis [12], [13], pregnancy disorders [14] and with various neurodegenerative pathologies such as dementia, Parkinson's and Alzheimer's diseases [15], [16], [17]. Indeed, cells have evolved mechanisms for the clearance of this toxic HTL. Urinary excretion of HTL in the kidneys [3], [18] and serum homocysteine-thiolactonase associated with high density lipoprotein which is known to hydrolyse HTL [19], form the extracellular mode of HTL clearance from the body, while bleomycin hydrolase (BHL) is the major intracellular HTL-hydrolysing enzyme which protects the cells against intracellular HTL [20]. However, under severe hyperhomocysteinemic conditions, the effectiveness of these mechanisms in protecting cells against HTL toxicity are still not yet investigated and properly understood.

Several studies have shown that one of the likely mechanisms underlying harmful effects of homocysteine (Hcy) is the chemical modification of protein by homocysteine thiolactone (HTL), a highly reactive cyclic thioester of Hcy [21], [22], [23], [24], which is formed by methionyl-tRNA synthetase [21], [24], [25] in an error editing reaction. It has been demonstrated that HTL preferentially forms amide bonds with *ε*-amino group of protein lysine residues in a non-enzymatic mechanism; a process referred to as “protein N-homocysteinylation” [19]. Protein N-homocysteinylation, has therefore, been considered to be one of the basic causes of HTL toxicity. Incorporation of HTL to the proteins is believed to result in loss of protein functions due to alterations in the protein structure and become susceptible to further damage by oxidation [22], [26], [27]. In addition, it has also been observed in few proteins that N-homocysteinylation induces protein aggregation or amyloid formation and hence considered to be an independent risk factor for neurodegenerative diseases in human [17], [22], [28], [29], [30], [31], [32]. It may be noted that a large fraction of the plasma proteins and many proteins from other sources have been identified for protein N-homocysteinylation both *in vivo* and *in vitro* [22], [33]. However, all the structural and functional based studies have been limited to very few proteins. Since, the total number of proteins modified is large and includes proteins having different physico-chemical properties and fold types, it is important to investigate the structural and functional consequences of HTL on proteins having different physico-chemical properties. In the present study, we have investigated the effects of HTL on three different proteins having different physico-chemical properties (namely lysozyme, RNase-A and alpha-LA). We found that N-homocysteinylation has opposite consequences on acidic and basic proteins suggesting that pI of the protein determines the extent of homocysteinylation, and the structural and functional consequences due to homocysteinylation. Most interestingly, basic proteins are resistant to the structural and functional loss due to N-homocysteinylation indicating that homocysteinylation does not necessarily lead to functional alterations.

## Materials and Methods

### Materials

Commercially lyophilized preparations of lysozyme (from chicken egg white), ribonuclease-A (RNase-A; from bovine pancreas), alpha-lactalbumin, alpha-casein (α-LA and CN respectively; from bovine milk) and carbonic anhydrase II (CA from bovine erythrocytes) were purchased from Sigma Chemical Co. DL-homocysteine thiolactone hydrochloride, 8-anilino-1-naphthalene sulfonic acid (ANS), thioflavin-T (ThT), 5, 5′-Dithiobis (2-nitrobenzoic acid), cytidine 2′-3′ cyclic monophosphate (C>p), *M. luteus* cells and p-nitrophenol acetate (pNPA) were also obtained from Sigma Chemical Co. Potassium chloride and potassium phosphate were obtained from Merck. Guanidinium chloride (GdmCl) was the ultrapure sample from MP Biomedicals. These and other chemicals, which are of analytical grade, were used without further purification.

Lysozyme, RNase-A, α-LA, CN and CA solutions were dialyzed extensively against 0.1 M KCl at pH 7.0 in cold (∼4°C). Protein stock solutions were filtered using 0.22 µm millipore syringe filters. Concentrations of the protein solutions were determined experimentally using *ε*, the molar extinction coefficient values of 39000 M^−1^cm^−1^ at 280 nm for lysozyme [34], 9800 M^−1^cm^−1^ at 277.5 nm for RNase-A [35], 29210 M^−1^cm^−1^ at 280 nm for α-LA [36], 11,000 M^−1^cm^−1^ at 280 nm for CN [37] and 57,000 M^−1^cm^−1^ at 280 nm for CA [38]. The concentration of GdmCl stock solution was determined by refractive index measurements [39]. All solutions for optical measurements were prepared in the appropriate degassed buffer. All experiments were carried out in 0.05 M potassium phosphate buffer (pH 7.4) containing 0.1 M KCl at 37°C.

### Protein N-homocysteinylation

All proteins (2 mg ml^−1^) were incubated in the presence of different concentrations of HTL (0–1000 µM) in 0.05 M potassium phosphate buffer, pH 7.4 overnight at 37°C. The HTL treated/untreated protein samples were further used for subsequent studies.

### Protein sulfhydryl estimation using Ellman's reagent

Protein sulfhydryl (SH) group estimation was carried out as described by Ellman [40] with some minor modifications. Briefly, fractions containing unmodified and modified proteins were solubilized in 6 M guanidinium hydrochloride in presence of 2 mM β-mercaptoethanol (ME) and incubated for 1 h at 37°C as described earlier [30], [31], [32]. Proteins were then precipitated down with 10% TCA to remove unbound HTL. Protein pellets were collected and resolubilized in phosphate buffer, pH 7.0. The levels of thiol groups in control and homocysteinylated protein samples were assayed using 5, 5′-Dithiobis (2-nitrobenzoic acid), the Ellman's reagent [40]. The absorbance of the samples was measured at 412 nm, using a 1 cm path-length cuvette. The amount of 5′-nitrothiobenzoate released was estimated from the molar extinction coefficient (*ε*) of 13,700 M^−1^ cm^−1^.

### Circular Dichroism (CD) Measurements

CD measurements (at least in triplicates) were made in a Jasco J-810 spectropolarimeter equipped with a Peltier-type temperature controller with six accumulations. Protein concentration used for the CD measurements was 0.5 mg ml^−1^. Cells of 0.1 and 1.0 cm path lengths were used for the measurements of the far- and near-UV CD spectra, respectively. Necessary blanks were subtracted for each measurement. All readings were procured at 37°C. The CD instrument was routinely calibrated with D-10-camphorsulfonic acid. Secondary structure estimation from the far-UV CD spectra was calculated using Yang's method [41].

### Fluorescence Measurements

Fluorescence spectra of the protein samples were measured in a Perkin Elmer LS 55 Spectrofluorimeter in a 3 mm quartz cell, with both excitation and emission slits set at 10 nm (at least in triplicates). Protein concentration for all the experiments was 2 µM for lysozyme; 5 µM for RNase-A, α-LA, CN and CA. For intrinsic fluorescence measurements, RNase-A was excited at 268 nm, while the emission spectra were recorded from 290–350 nm. Lysozyme, α-LA, CN and CA were excited at 295 nm and the emission spectra were recorded in the wavelength region 300–450 nm.

For ANS-protein binding experiments, the excitation wavelength was 360 nm, and emission spectra were recorded from 400–600 nm. ANS concentration was kept 16 fold that of protein concentration. For ThT-protein binding experiments, the excitation wavelength used was 450 nm, and emission spectra were recorded from 475–570 nm. ThT concentration was kept 25 µM. Necessary blanks were subtracted for each sample. Each spectrum was repeated at least three times.

### Transmission electron microscopy

Modified protein solutions were placed on a copper grid and left at room temperature for 5 min. For negative staining of the samples, 1.0% uranyl acetate solution was added on to the copper grid and allowed to air dry before examination using a FEI Tecnai G2-200kV HRTA transmission electron microscopy (Netherland) operating at 200 kV.

### Dynamic light scattering measurements

Size distribution of the particles present in the protein sample were obtained using a Zetasizer Micro V/ZMV 2000 (Malvern, UK). Measurements were made at a fixed angle of 90° using an incident laser beam of 689 nm. Fifteen measurements were made with an acquisition time of 30 seconds for each sample at sensitivity of 10%. The data was analysed using Zetasizer software provided by the manufacturer to get hydrodynamic diameters. The protein concentration was 2.0 mg ml^−1^. All measurements were performed at 37°C.

### Activity measurements

For measuring lysozyme activity, we used *M. luteus* cells as substrate and followed the procedure of Maurel and Douzou [42]. The reaction was followed in Jasco V- 660 UV/Visible spectrophotometer. We observed that the apparent specific absorbance (the slope of the straight line obtained by plotting the decrease in absorbance at 450 nm against concentration of the substrate in the range 0–150 mg l^−1^) of an aqueous suspension of *M. luteus* cells was *ε*_450_ = 0.65×10^−2^ mg l^−1^. Just before the initiation of the enzymatic reaction, a given concentration of the substrate in the buffer was transferred to sample and reference cuvettes which was kept at 37.0±0.1°C and allowed to equilibrate for 15 min. In order to follow the progress curve, 25 µl of lysozyme from the stock of 2 mg ml^−1^ was added in the sample cuvette by rapid mixing. To reflect the same dilution, 25 µl of buffer was also added in the reference cuvette. The decrease in absorbance, which occurred during the lysis of the cell wall, was recorded at 450 nm for 20 min. The initial velocity (*v*) of lysis was deduced from the slope of the linear part of the recordings, usually over 30 s. This experiment was repeated for different concentrations of the substrate in the range 10–150 mg ml^−1^, and a plot of v versus [S] (in mg l^−1^) was generated. The plot of *v* versus [S] was analyzed for *K*_m_ and *V*_max_ using the relation (Equation 1),
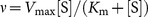
(1)where *v* is the initial velocity, and [S] is the substrate concentration. From this analysis the values of *k*_cat_ were determined.

In order to see the effect of a HTL on the kinetic parameters (*K*_m_ and *k*_cat_) of lysozyme, the substrate and the enzyme were pre-incubated in a given concentration of the HTL. Reaction at each concentration of the HTL was followed exactly the same way as described for the control experiment.

Following the procedure described by Crook *et al.* [43], RNase-A activity using cytidine 2′-3′ cyclic monophosphate (C>p) as a substrate was measured. Progress curve for RNase-A mediated hydrolysis of C>p in the concentration range (0.05–0.50 mg ml^−1^) in the absence and presence of a given concentration of a HTL was followed by measuring change in absorbance at 292 nm for 20 min in Jasco V-660 UV/Vis spectrophotometer. Sample and reference cells were maintained at 37.0±0.1°C. From each progress curve at a given substrate concentration and in the absence and presence of a fixed HTL concentration, initial velocity (*v*) was determined from the linear portion of the progress curve, usually 30 s. The plot of initial velocity (*v*) versus [S] (in mM) at each HTL concentration was analyzed for *K*_m_ and *k*_cat_ using Eq. (1).

### Thermal Denaturation Studies

Thermal denaturation studies were carried out in a Jasco V- 660 UV/Visible spectrophotometer equipped with a Peltier-type temperature controller at a heating rate of 1°C per minute. This scan rate was found to provide adequate time for equilibration. Each sample was heated from 37 to 85°C. The change in absorbance with increasing temperature was followed at 287 nm for RNase-A, 300 nm for lysozyme. About 500 data points of each transition curve were collected. Measurements were repeated three times. After denaturation, the protein sample was immediately cooled down to measure reversibility of the reaction. Each heat-induced transition curve was analyzed for *T*_m_ (midpoint of denaturation) using a non-linear least squares method according to the relation (Equation 2),
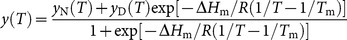
(2)where *y*(*T*) is the optical property at temperature *T* (Kelvin), *y*_N_(*T*) and *y*_D_(*T*) are the optical properties of the native and denatured protein molecules at *T* K, respectively, and *R* is the gas constant. In the analysis of the transition curve, it was assumed that a parabolic function describes the dependence of the optical properties of the native and denatured protein molecules (i.e. *y*_N_(*T*) = *a*_N_+*b*_N_ *T*+*c*_N_ *T* ^2^ and *y*_D_(*T*) = *a*_D_+*b*_D_ *T*+*c*_D_ *T* ^2^, where *a*_N_, *b*_N_, *c*_N_, *a*_D_, *b*_D_, and *c*_D_ are temperature-independent coefficients) [44].

## Results

**Figure 1:**
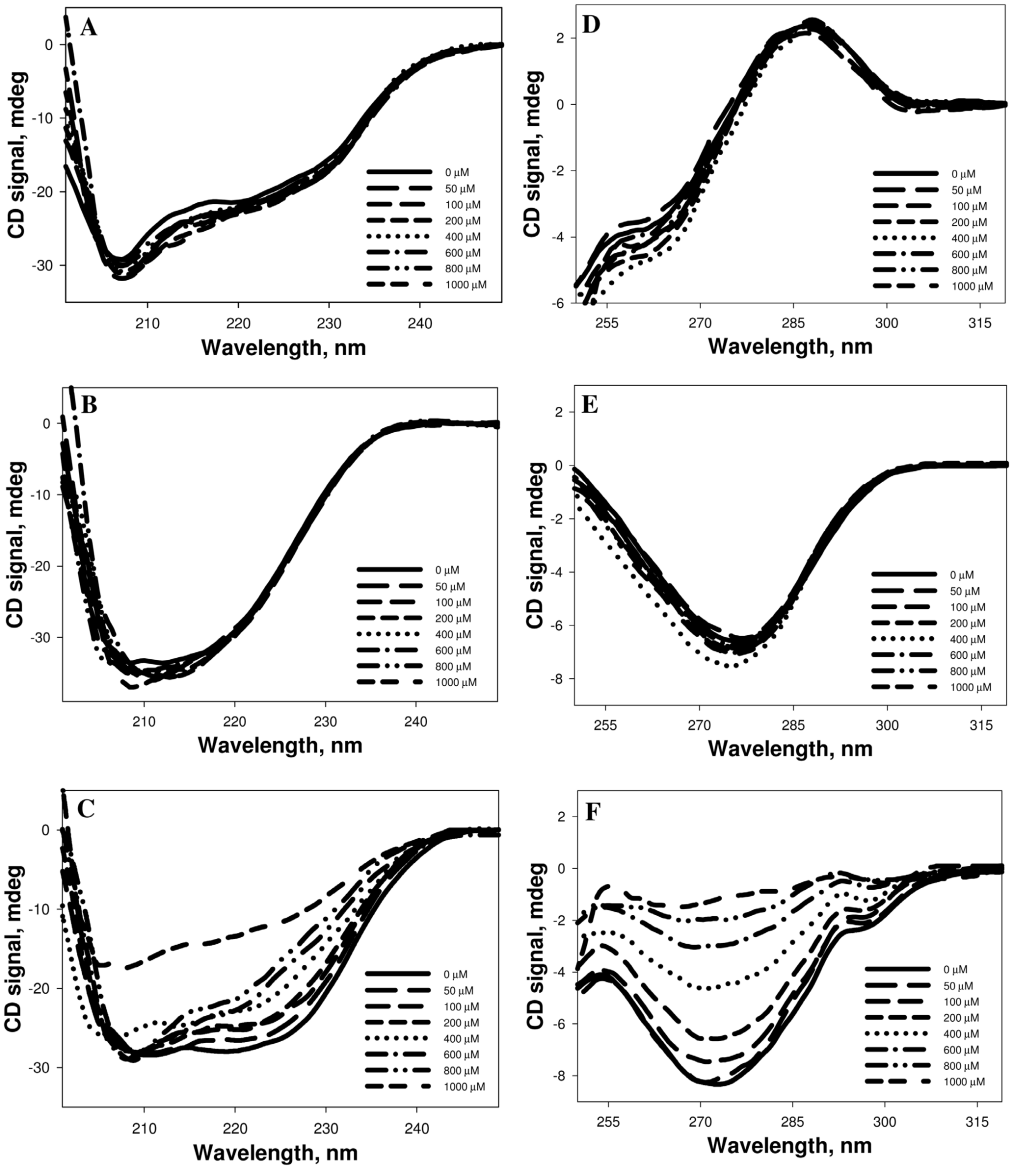
Circular dichroism spectra of homocysteinylated proteins. Far-UV CD spectra (at 37°C) of lysozyme (A), RNase-A (B) and α-LA (C) modified with varying concentrations of HTL ranging from 0–1000 µM, at pH 7.4 (left panel). Near-UV CD spectra of lysozyme (D), RNase-A (E) and α-LA (F) modified with varying concentrations of HTL ranging from 0–1000 µM, at pH 7.4 (right panel).

**Figure 2:**
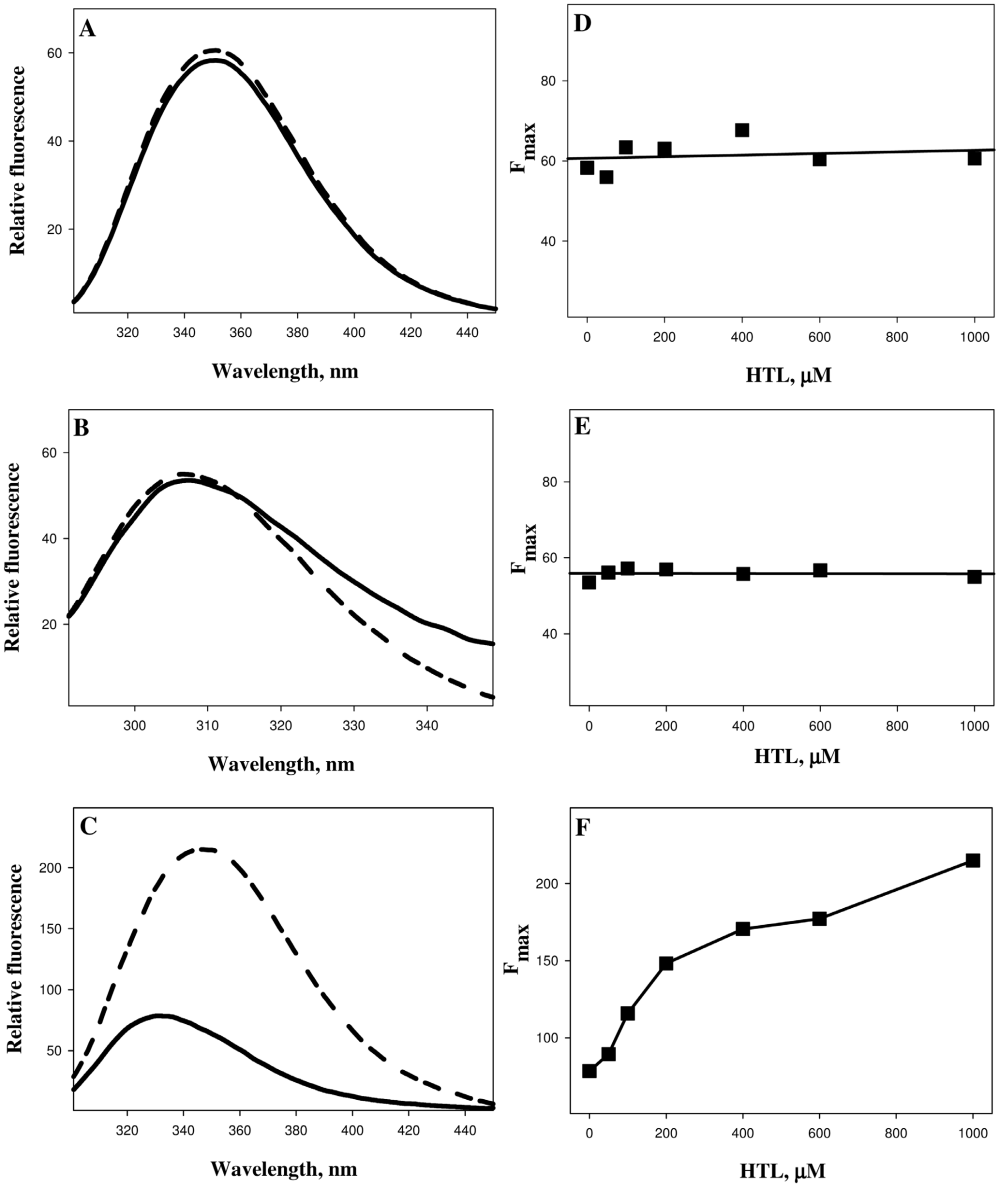
Intrinsic fluorescence spectra (left panel) of lysozyme (A), RNase-A (B) and α-LA (C) modified with HTL. To maintain clarity, only the representative curves of control unmodified protein (solid lines) and protein modified with 1000 µM HTL (dashed lines) are shown. Plots of the fluorescence maxima (F_max_) as a function of HTL concentrations of lysozyme (D), RNase-A (E) and α-LA (F) are given in the right panel.

To investigate the effects of N-homocysteinylation on proteins, we have chosen lysozyme, RNase-A and α-LA. The choice of the proteins was to have different isoelectric points (pI) and fold types keeping in mind that the proteins should also have different lysine contents (See Table 1). To modify proteins by HTL, each of the protein samples (2 mg ml^−1^) was treated with different concentrations of HTL, ranging from 0–1000 µM (incubated overnight at pH 7.4) and analyzed for the free SH contents using Ellman's reagent (See Table 2). HTL has been shown to be quite stable at room temperature with less than 10% degradation after 24 hours under physiological conditions [18], [21], [45], [46], [47], [48]. Hence, overnight (12–15 hrs) incubations would thereby lead to minimal hydrolysis of HTL. It is seen in Table 2 that the proteins have been incorporated with HTL as suggested by large increase in the free SH contents. Fig. 1 shows far-UV CD (left panel) and near-UV CD (right panel) spectra of the three HTL-modified proteins. This figure indicates that HTL-induced modification has different structural consequences on α-LA relative to lysozyme and RNase-A, in terms of secondary (far-UV CD) and tertiary (near-UV CD) structures. We further confirmed the alterations in the tertiary structure of the proteins due to the modification by measuring tryptophan/tyrosine fluorescence spectra of the homocysteinyalated proteins. Fig. 2 (left panel) shows representative tryptophan/tyrosine fluorescence spectra of the modified and unmodified proteins and the right panel shows the observed fluorescence maxima (F_max_) at different concentrations of HTL.

**Table 1:** Characteristics of lysozyme, RNase-A, α-LA, CN and CA.

	Lysozyme	RNase-A	α-LA	CN	CA
Molecular weight (kDa)	14.3	13.7	14.2	23	29
Number of Amino acids	129	124	123	214	260
pI	11.3	9.6	4.5	4.2	5.9
Fold type	α	αβ	α	IDP*	β
Secondary Structure (%)					
α-helix	30	21	26	18	21
β-sheet	13	39	15	26	36
No. of lysine residues	6	10	12	14	18

* The abbreviation of IDP is intrinsically disordered protein.

**Table 2:** Protein free sulfhydral contents on modification by HTL.^a^^,^ b

HTL (µM)	Lysozyme	RNase-A	α-LA
0	35.78	34.66	36.91
50	45.78	68.48	79.16
100	72.02	91.19	203.65
200	103.31	115.45	325.84
400	163.82	204.16	515.62
600	278.82	331.21	597.19
800	422.86	475.39	833.15
1000	547.74	609.27	935.56

a Errors sulfhydral contents are 5–8%.

b Unit of sulfhydral contents is (µM mg^−1^).

**Figure 3:**
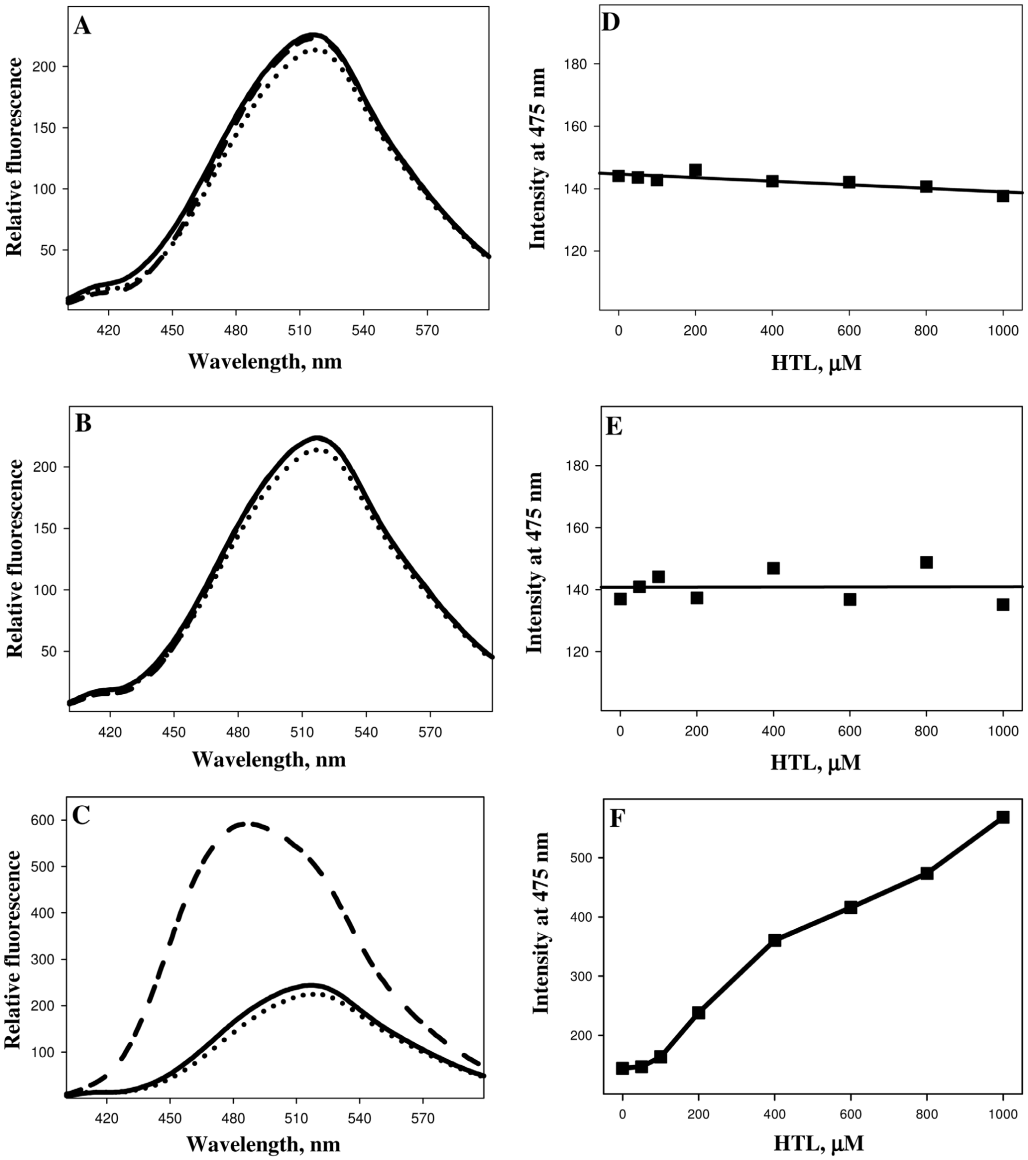
ANS binding assay (left panel) of lysozyme (A), RNase-A (B) and α-LA (C) treated overnight at 37°C with varying concentrations of HTL. Right panels depict λ_max_ as a function of HTL concentrations of lysozyme (D), RNase-A (E) and α-LA (F). To maintain clarity, only the representative curves of free dyes (dotted lines), control unmodified protein (solid lines) and protein modified with 1000 µM HTL (dashed lines) are shown.

**Figure 4:**
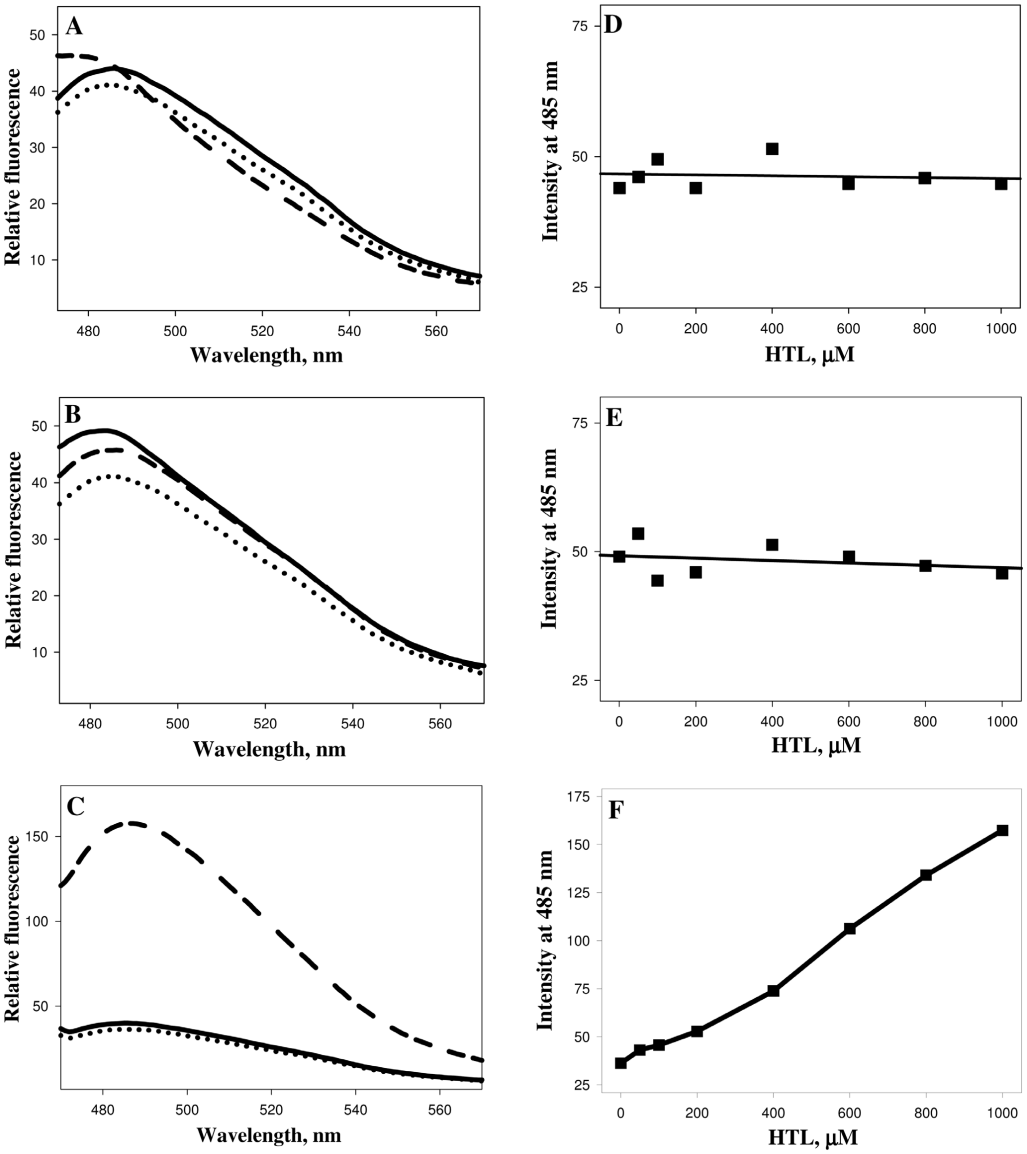
ThT binding assay (left panel) of lysozyme (A), RNase-A (B) and α-LA (C) treated overnight at 37°C with varying concentrations of HTL. Right panels depict λ_max_ as a function of HTL concentrations of lysozyme (D), RNase-A (E) and α-LA (F). To maintain clarity, only the representative curves of free dyes (dotted lines), control unmodified protein (solid lines) and protein modified with 1000 µM HTL (dashed lines) are shown.

**Figure 5:**
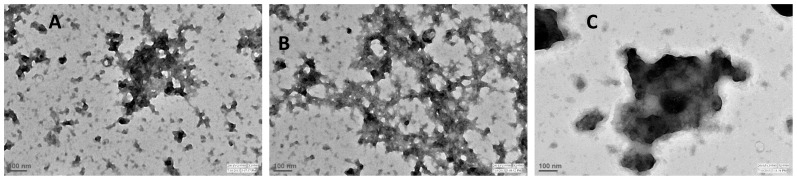
Transmission electron micrographs of α-LA. Micrographs of α-LA samples incubated overnight with HTL at 37°C (A-400 µM, B-600 µM and C-1000 µM HTL). See also S1 Fig.

We then performed ANS and ThT binding assays to investigate if N-homocysteinylation induces aggregate formation of the HTL-modified proteins (Fig. 3 and Fig. 4). It was observed that there is no binding of both ANS and ThT in case of lysozyme and RNase-A at different concentrations of HTL, as neither the λ_max_ was blue shifted (for ANS) nor there is an increase in the relative fluorescence intensities for both ANS and ThT. Whereas in the case of α-LA there is an increase in both the ANS and ThT binding in an HTL concentration dependent manner upon modification by HTL indicating the presence of protein aggregates that might be amyloidogenic. Transmission electron microscopy images (Fig. 5) further confirm that N-homocysteinylation-induced aggregates in α-LA are not amyloidogenic in nature. S1 Fig. shows the size distribution by volume and Table 3 shows the hydrodynamic diameter of protein aggregates obtained from DLS measurements. It is seen in S1 Fig. that there is existence of large aggregates due to modification. The results together indicate that protein covalent modification by N-homocysteinylation has different consequences in terms of structure and aggregation propensities on different proteins.

**Table 3:** Hydrodynamic diameter of HTL-treated α-LA samples.

HTL (µM)	Hydrodynamic diameter (nm)
0	3.6±.3
50	10.6±.8
100	36.3±1.5
200	107.9±8.7, 389.8±17.9
400	155.7±8.9, 342.8±39.6
600	198.3±10.3, 941.7±78.3
800	225.6±23.2, 1044.0±65.5
1000	428.3±34.1, 1491.0±163.4

Solution containing 2 mg ml^−1^ of α-LA was withdrawn from the reaction mixtures and analysed without any dilution. All measurements were performed at 37°C. The values reported represent the mean ± SD calculated from three independent experiments.

Following the procedure described in the preceding section, functional activity parameters (*K*_m_ and *k*_cat_) of lysozyme and RNase-A were measured in the absence and presence of different concentrations of HTL at pH 7.4 and 37°C. Table 4 shows the enzyme kinetic parameters of HTL-modified (and -unmodified) lysozyme and RNase-A. It is seen in this table that there is no significant effect on the functional activity parameters (*K*_m_ and *k*_cat_) of lysozyme and RNase-A due to the modification. No changes in the *K*_m_ and *k*_cat_ might possibly be due to no affects in the thermodynamic stability of the modified proteins. To uncover this possibility, we performed heat-induced denaturation studies of the modified lysozyme and RNase-A by monitoring the changes in absorbance at 300 nm for lysozyme and 287 nm for RNase-A as a function of temperature. The denaturation profiles are shown in S3 Fig. and the measured thermodynamic parameter (*T*_m_) are presented in Table 5. It should however be noted that a complete transition curve could not be obtained in the temperature range of 37°C–85°C in the case of lysozyme. Therefore, in order to bring down transition curves in the measurable temperature range, 1.5 M GdmCl was added to the samples in case of lysozyme. Therefore, the transition curves shown in S3 Fig. for lysozyme (upper panel) is the curve obtained in the presence of 1.5 M GdmCl. The unfolding profiles are given in S3 Fig. and the evaluated *T*_m_ using appropriate equation (Equation 2, see materials and methods) are given in Table 5. It is seen in S3 Fig. (and Table 5, for *T*_m_), that there is no significant change in protein stability in terms of *T*_m_. Our results clearly indicate that N-homocysteinylation brought about by HTL does not affect the functional activities of lysozyme and RNase-A by not affecting the thermodynamic stability, *T*_m_.

**Table 4:** Enzymatic activity parameters (*K*_m_ and *k*_cat_) of lysozyme and RNase-A treated with different concentrations of HTL^a^.

	Lysozyme		RNase-A	
HTL (µM)	*K*_m_ (µg ml^−1^)	*k*_cat_ (mg ml^−1^ s^−1^M^−1^)	*K*_m_ (mM)	*k*_cat_ (min^−1^)
0	77.8±2	484.1±29	1.29±0.11	191±22
50	76.9±4	489.3±25	1.33±0.17	185±23
100	78.1±3	479.9±27	1.27±0.09	192±25
200	77.4±4	481.3±31	1.28±0.13	195±28
400	77.6±3	478.8±26	1.29±0.12	194±23
600	77.1±2	482.3±24	1.31±0.14	196±27
800	78.3±3	484.5±28	1.32±0.16	189±22
1000	77.3±4	481.2±31	1.25±0.13	198±24

a ± represents the average errors of three independent measurements.

**Table 5:** Melting temperature (*T*_m_) of lysozyme and RNase-A on being treated overnight with HTL at 37°C^a^.

HTL (µM)	Lysozyme	RNase-A
0	67.6	63.4
50	67.4	63.5
100	67.8	63.1
200	68.1	63.4
400	68.3	63.3
600	67.6	63.4
800	67.1	62.9
1000	67.3	63.1

a Errors in *T*_m_ are .2–1%. Measurements were repeated at least three times.

## Discussion

**Figure 6:**
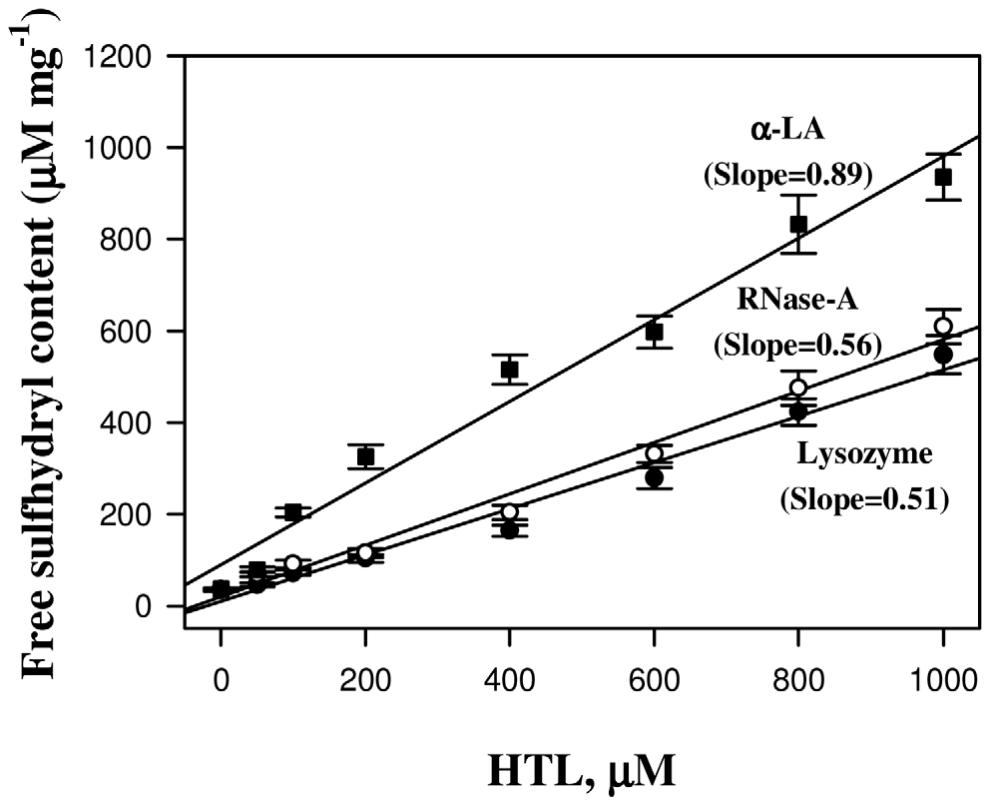
A comparative analysis for the extent of N-homocysteinylation as assessed by protein free sulfhydryl estimation using Ellman's reagent. Filled circles, open circles and filled squares represent lysozyme, RNase-A and α-LA, respectively.

**Figure 7:**
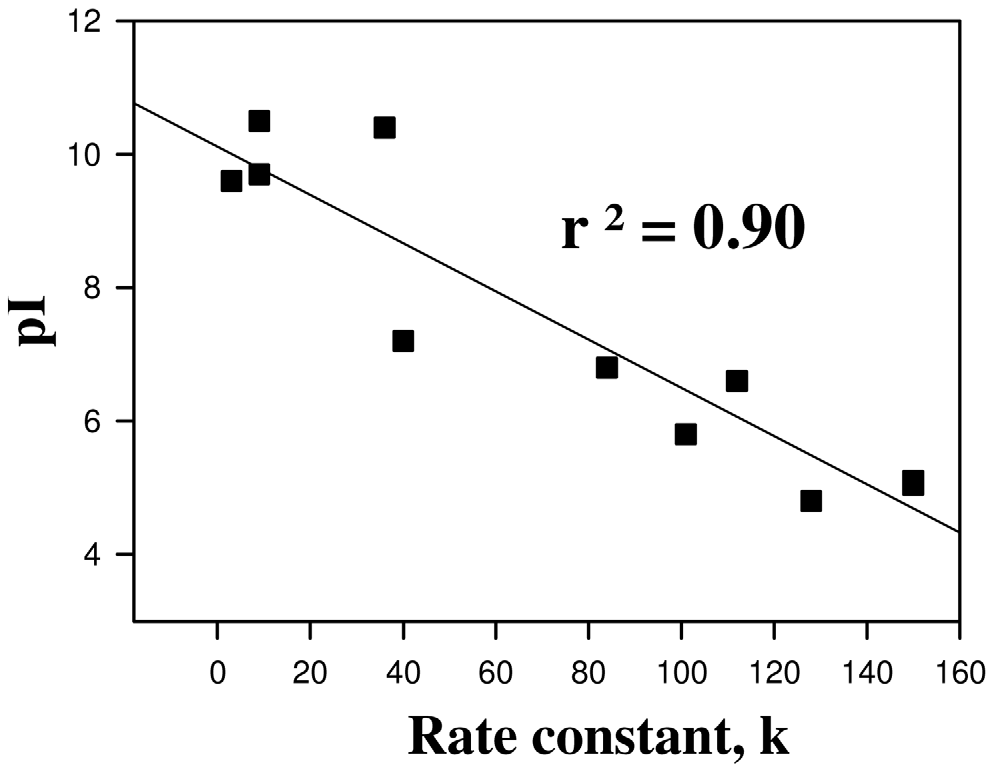
Relationship between proteins pI and second-order rate constants (k) of their modification by HTL. Second-order rate constants (k) are plotted as a function of the pI for the following proteins: low density lipoproteins, transferrin, albumin, γ-globulin, fibrinogen, hemoglobin, myoglobin, cytochrome c, α-crystallin, trypsin, DNase-I and RNase-A (in order of increasing pI). Values are taken from 22.

It has been previously reported that protein N-homocysteinylation basically targets the free amino group of lysine residues in protein. Therefore, first of all we have investigated if there is any difference in the extent of homocysteinylation among the proteins (lysozyme, RNase-A and α-LA) having different lysine residues (see Table 1). The HTL concentrations (0–1000 µM) used in the present study have been selected keeping in mind that these also represent a near pathological concentrations of Hcy found in severe homocysteinuric conditions [7], [8], [9], [10], [11]. Covalent adduct formation between HTL and protein lysine residues results in the availability of a free SH in the modified protein. Since for each HTL molecule reacting with the free amino group of the protein, an SH group is added which can be assessed using Ellman's reagent. Hence increase in the free SH content upon HTL treatment is regarded to be a good signature of protein covalent adduct formation by HTL. It is seen in Table 2 that there is an increase in the free SH content of the proteins in a HTL concentration manner suggesting that the proteins have been incorporated with HTL. Interestingly, the extent of homocysteinylation is almost same in the case of lysozyme and RNase-A, but is highest in case of α-LA (Fig. 6). In terms of number of lysine residues present in the proteins, RNase-A and α-LA should have almost the similar extents of homocysteinylation (as the number of lysine content does not vary much, RNase-A and α-LA have 10 and 12 lysine residues respectively); while lysozyme and RNase-A should have different extent of homocysteinylation as the number of lysine content in RNase-A is relatively higher (See Table 1). In fact, the dependence of the extent of homocysteinylation on the number of lysine content in proteins has been challenged by several studies [28], [30], [49], [50]. Since, both lysozyme and RNase-A are basic proteins with close pI values and α-LA is an acidic protein (See Table 1), we speculated that the differential behaviour of N-homocysteinylation may most probably be due to the differences in their pI values (or the acidic or basic nature) of the proteins. Several serum proteins have been found to have modified with HTL and their rate of N-linked modification due to HTL has also been reported [22]. It may be noted that most serum proteins reported for N-linked homocysteinylation were found to have low pI values. In order to see if pI values might play a role in the N-homocysteinylation of proteins, we have plotted the rate of homocysteinylation as a function of pI for all the serum proteins reported to have modified by homocysteinylation (Fig. 7). Fig. 7 shows that there is a close relation between the pI and the rate of homocysteinylation (k). The existence of such a close relation between the pI and the k values led us to believe that the acidic proteins are prone to N-homocysteinylation while basic proteins tend to have lesser extent of homocysteinylation.

It has been known that protein N-homocysteinylation results in native state structural alterations, loss of enzyme functions and induces protein aggregation/amyloidogenesis [11], [22], [28], [29], [30], [31], [32], [51], [52], [53]. Therefore, it is important to investigate if these consequences of protein homocysteinylation depend on the acidic or basic nature of the proteins. In this spirit, we have investigated if acidic and basic proteins behave differently on being modified by N-homocysteinylation. We have investigated if any difference in the native state conformational changes due to homocysteinylation is responsible for the differential behavior of homocysteinylation on the proteins. For this, we have measured the far-UV, near-UV CD and intrinsic fluorescence spectra of the homocysteinylated proteins. It is seen in Fig. 1 that the secondary structural contents of lysozyme and RNase-A are not changed due to the modification at different concentrations of HTL. In contrast, the secondary structure of α-LA has been reduced significantly in a HTL concentration dependent manner and completely denatured at the highest concentration of HTL (1000 µM). Evaluated percent secondary structural changes (see Table 6) also suggest that there is no alterations in alpha and beta content of lysozyme and RNase-A samples treated with HTL. However, in the case of α-LA, we observed a decrease in the percent alpha helical content and a concomitant increase in the percent beta sheet structural composition. Thus, N-linked modification of α-LA by HTL brings about structural transition from an alpha to beta sheets but this conversion is absent in the case of lysozyme and RNase-A. Near-UV CD and intrinsic fluorescence spectral measurements of the modified lysozyme and RNase-A at different HTL concentrations also indicate that there is no tertiary structural change due to modification. In case of α-LA, HTL binding induces alterations in the tertiary structure leading to complete loss of tertiary structure at the highest concentration of HTL. In addition to lysozyme and RNase-A used in the present study, many proteins have earlier been reported to be N-homocysteinylated but do not result in structural alterations [49], [51]. Conformational measurements therefore indicate that the differential extent of N-homocysteinylation on acidic (α-LA) and basic (lysozyme and RNase-A) proteins is due to the different effects on the native states of the proteins. At present, we have no concrete explanation for the different effects of HTL-induced modifications on the native states of different proteins. It might, however be possible that disruption of the tertiary contacts due to N-homocysteinylation might be limiting step as the tertiary structures of both lysozyme and RNase-A could not be disrupted due to the modification. Perhaps the incorporation of HTL to specific lysine residues in different proteins might be responsible for the opening of the tertiary structure which in case of α-LA is easily accessible while it is difficult to target in case of lysozyme and RNase-A. In support of our argument, it has been previously reported in cytochrome c (cyt c), that modification of four lysine residues (Lys8 or -13, Lys86 or -87, Lys99, and Lys100) does not bring about any significant change in its native state tertiary structure [49]. However, a single residue modification in case of insulin results in denaturation, ultimately leading to aggregate formation [28], [54]. In addition, different reactivities of lysine residues in hemoglobin toward HTL were observed in another study [50]. Furthermore, it has been shown that Lys525 is a predominant site of *N*-homocysteinylation in case of human serum albumin and the status of Cys34 determines the reactivity of albumin lysine residues, including Lys525 [55]. All these results provide clear evidence that modification of specific lysine residues is responsible for the observed structural and hence functional consequences.

**Table 6:** Percent secondary structure of lysozyme, RNase A and α-LA on modification by HTL.

	Lysozyme		RNase-A		α-LA	
HTL (µM)	α-helix	β-sheet	α-helix	β-sheet	α-helix	β-sheet
0	27.8	14.6	20.6	40.6	28.9	13.9
50	27.3	13.8	21.1	42.1	27.1	16.2
100	28.6	12.6	19.7	41.6	22.8	18.8
200	28.7	14.1	20.2	42.5	21.5	20.7
400	29.2	14.4	19.8	39.3	16.0	28.3
600	27.9	13.8	19.1	41.7	15.3	30.7
800	29.4	15.1	21.7	40.9	13.7	32.9
1000	28.7	14.8	20.6	40.5	11.3	36.4

Secondary structure estimation was accomplished according to Yang's method (41).

We have further investigated if all of the homocysteinylated proteins used in this study undergo aggregate formations. For this purpose we analysed the homocysteinylated proteins for ANS and ThT binding propensities. The absence of ANS and ThT binding in lysozyme and RNase-A indicate no possibility of the presence of aggregates in the protein samples treated with HTL. Interestingly the extensively homocysteinylated protein, α-LA (as compared to lysozyme and RNase-A) was found to have a very prominent binding of both ANS and ThT indicating the presence of non-native aggregates which might be most probably amyloids. In addition, DLS measurement suggests the presence of at least two different aggregate species at concentration of HTL beyond 100 µM (Table 3). On being modified with HTL the hydrodynamic diameter of α-LA is highly increased with predominant particle size of ∼1500 nm (at 1000 µM) as compared to 3.56 nm of the control native proteins suggesting that at this concentration of HTL, the monomeric native α-LA has completely disappeared. To further confirm if the aggregates formed are amyloidogenic in nature, we have analyzed them using transmission electron microscopy, which revealed the presence of amorphous structures but not fibrils. Binding of ThT, but absence of fibrils/amyloids might possibly mean that the native α-LA has changed from its alpha helical conformation to predominant beta rich conformations that do not have the propensity to form fibrils. It may be noted that the HTL-induced amyloid formation has so far been reported only on very few proteins [30], [32]. Hence N-homocysteinylation-induced amyloid transformation might not be a general consequence on all proteins.

To examine the generality of the dependence of HTL-induced structural alterations on acidic proteins, we performed similar measurements on two additional acidic proteins (α-casein, CN, pI 4.2 and carbonic anhydrase, CA, pI 5.9). Similar to α-LA, it was also found that HTL induces structural alterations in CN and CA, as evident from the changes in the intrinsic fluorescence emission maxima on being treated with HTL and binding of ANS and ThT (See Figs. S2, S4). In agreement to the observed aggregate formation on α-LA, CN and CA, many acidic proteins have been reported to form large oligomers to aggregates upon modification by HTL [28], [29], [30], [32], [52]. Thus it is clear from the results that in contrast to the observed effect on the basic proteins (lysozyme and RNase-A), N-homocysteinylated acidic proteins are unfolded leading to aggregate formation.

We have further investigated the effect of N-homocysteinylation on the functional activity of the two basic proteins (lysozyme and RNase-A). For this, we carried out measurement of enzymatic kinetic parameters (*K*_m_ and *k*_cat_) of HTL-modified lysozyme and RNase-A (see Table 4). It should be noted that the value for each kinetic parameter given in Table 4 represents the mean of three independent measurements and ± represent the mean error. The kinetic parameters in the absence of the HTL, shown in Table 4, are in excellent agreement with the previous reports [42], [43]. This agreement led us to believe that our measurements of the enzyme-catalyzed reactions and the analysis of the progress curves for kinetic parameters are accurate and authentic. Interestingly, it is also seen in Table 4 that the *K*_m_ and *k*_cat_ of the homocysteinylated lysozyme and RNase-A are not altered significantly due to N-homocysteinylation suggesting that the enzyme activity is not changed. However, in the case of CA which is an acidic protein, at 1000 µM HTL concentration, there was almost complete loss of enzyme activity (see S5 Fig.). No alterations in the thermodynamic stability (in terms of *T*_m_, see S3 Fig. and Table 5) due to homocysteinylation in lysozyme and RNase-A further support the fact that the protein functional activity should not be perturbed as protein stability and activity has a direct relation. It is now important to highlight that the commonly held belief of the loss of enzyme activity due to homocysteinylation has been derived from availability of large amount of N-homocysteinylated protein in serum of homocysteinuric patients and the existence of antibodies against these protein adducts, but not based on direct enzymatic activity measurements. However, based on systematic activity measurements, at least four proteins (namely NOS: nitric oxide synthase, MetRS: methionyl-tRNA synthetase, DDAH: dimethylarginine dimethylaminohydrolase and paroxonase) have been reported to result in loss of enzyme activity due to homocysteinylation [22], [51], [53], [56], [57]. All of these proteins reported were found to be acidic in nature with pI values ranging from 5.1–6.2. Thus considering all previous reports and this study, we conclude that N-homocysteinylation-induced protein modifications might be pI dependent, with acidic proteins having higher propensities for structural and functional alterations as compared to basic proteins.

Misfolded protein aggregates and plaques in neuronal cells are emblematic signature of most of the neurological disorders and can trigger cascade of events ultimately resulting in synaptic dysfunction and consequent neuronal death with devastating clinical consequences, and one of the basic symptoms of increased levels of Hcy is neurodegeneration. It is important to note that almost all cytoskeletal proteins are acidic in nature. The possibility of these cytoskeletal proteins getting homocysteinylated is quite high, resulting in loss of their structure and function. This will eventually hamper intracellular transport and trafficking systems, thereby resulting in reduced/loss of neuronal activity. To date, at least two proteins of cytoskeletal origin (microtubule-associated tau protein, neuronal and glial intermediate filaments, IF) [58] have been reported to be affected by increased level of Hcy. N-homocysteinylation of tau protein has been shown to result in altered tubulin assembly dynamics *in vitro* [59]. These results clearly indicate that cells or tissues rich in acidic proteins could be the major targets for Hcy induced cytotoxicity and hence a prime cause of neurodegeration. Taken together, we conclude that N-homocysteinylation-induced loss of protein functions is not generally true and may depend on the physico-chemical properties of the proteins.

## Conclusions

We provide here for the first time that the structural and functional consequences due to N-homocysteinylation depend on pI of the proteins. Basic proteins are resistant to structural and functional alterations due to N-homocysteinylation, whereas acidic proteins result in denaturation and ultimately leading to aggregation. The study indicates that not all proteins are prone to HTL-induced structural and functional modifications. In addition, cells or tissues rich in acidic proteins could be the major targets for Hcy-induced cytotoxicity.

## Supporting Information

S1 Fig **DLS analysis of both untreated and HTL-treated α-LA sample.** Size distribution by volume of control untreated sample (upper panel) and α-LA treated with 1000 µM HTL (lower panel).
(TIF)

S2 Fig **Fluorescence spectra of CN modified with varying concentrations of HTL.** Left panel depicts intrinsic fluorescence (A), ANS binding assay (B) and ThT binding assay (C). Right panel depicts F_max_ as a function of HTL concentration (D), and λ_max_ as a function of HTL concentrations (E and F). To maintain clarity, only the representative curves of free dyes (dotted lines), control unmodified protein (solid lines) and protein modified with 1000 µM HTL (dashed lines) are shown.
(TIF)

S3 Fig **Representative thermal denaturation profiles of lysozyme and RNase-A.** Denaturation curves of lysozyme (upper panel) and RNase-A (lower panel), in the absence and presence of various HTL concentrations at pH 7.4 phosphate buffer.
(TIF)

S4 Fig **CD and fluorescence spectra of HTL-modified CA.** Far UV-CD (A), intrinsic fluorescence spectra (B), ANS binding assay (C) and D-ThT binding assay (D) of HTL-modified CA. All spectra have been made in absence (solid lines) and in presence of 1000 µM HTL (dashed lines). In case of ANS and ThT binding assays, free dyes (dotted lines) have also been shown.
(TIF)

S5 Fig **Effect of HTL-induced modification on CA enzymatic activity.** Enzyme activity (in percent) was measured by monitoring the hydrolysis of p-nitrophenyl acetate (pNPA) at 400 nm. The enzyme concentration used was.03 mg ml^−1^ and substrate was kept 1 mM.
(TIF)
