# Supplementary figures and images for: N-Homocysteinylation Induces Different Structural and Functional Consequences on Acidic and Basic Proteins
Source: PLoS One. 2014 Dec 31;9(12):e116386. doi: 10.1371/journal.pone.0116386 (PMC4281231; doi:10.1371/journal.pone.0116386)

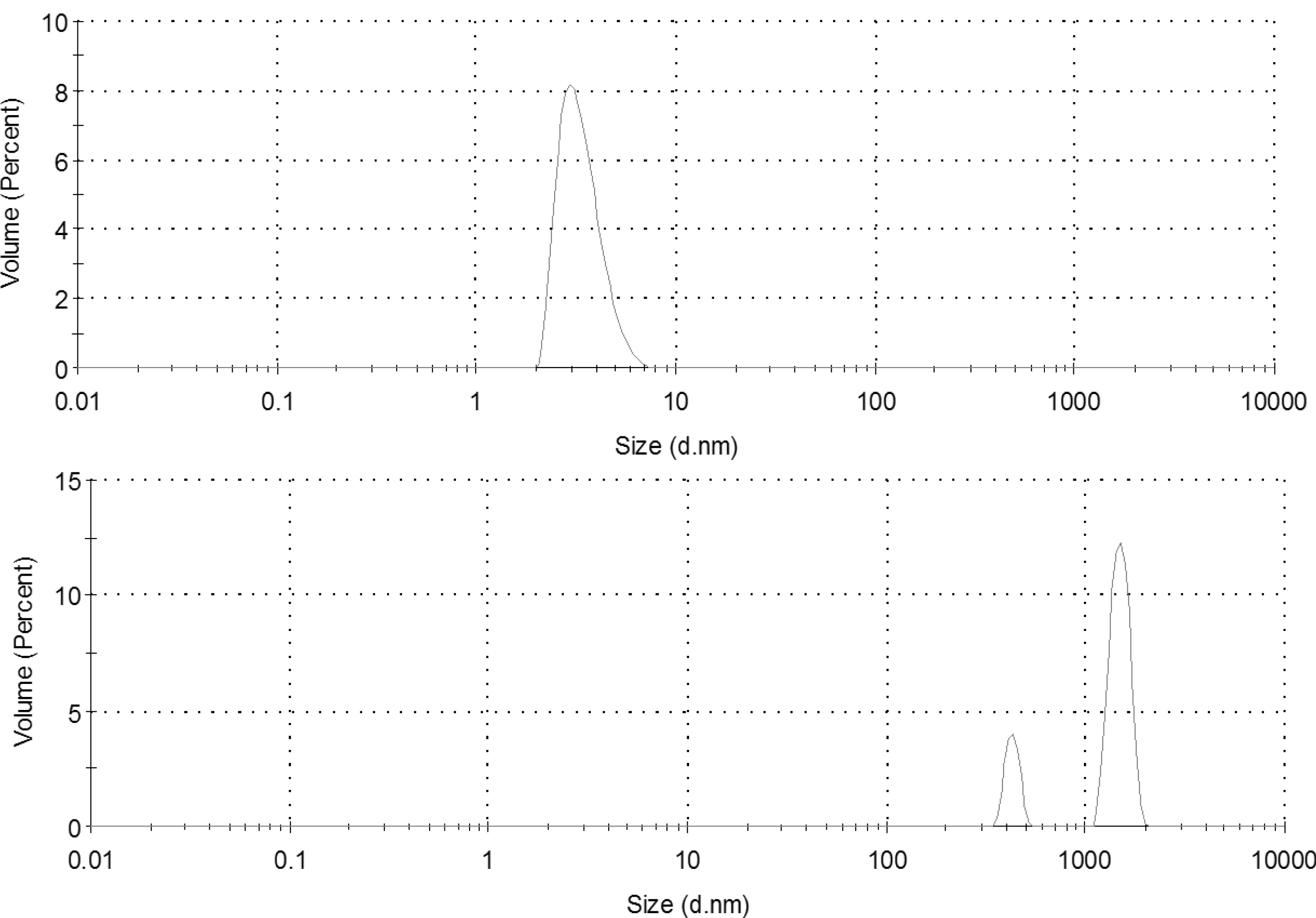

Supplement: S1 Fig — DLS analysis of both untreated and HTL-treated α-LA sample. Size distribution by volume of control untreated sample (upper panel) and α-LA treated with 1000 µM HTL (lower panel). (TIF) [file pone.0116386.s001.tif]

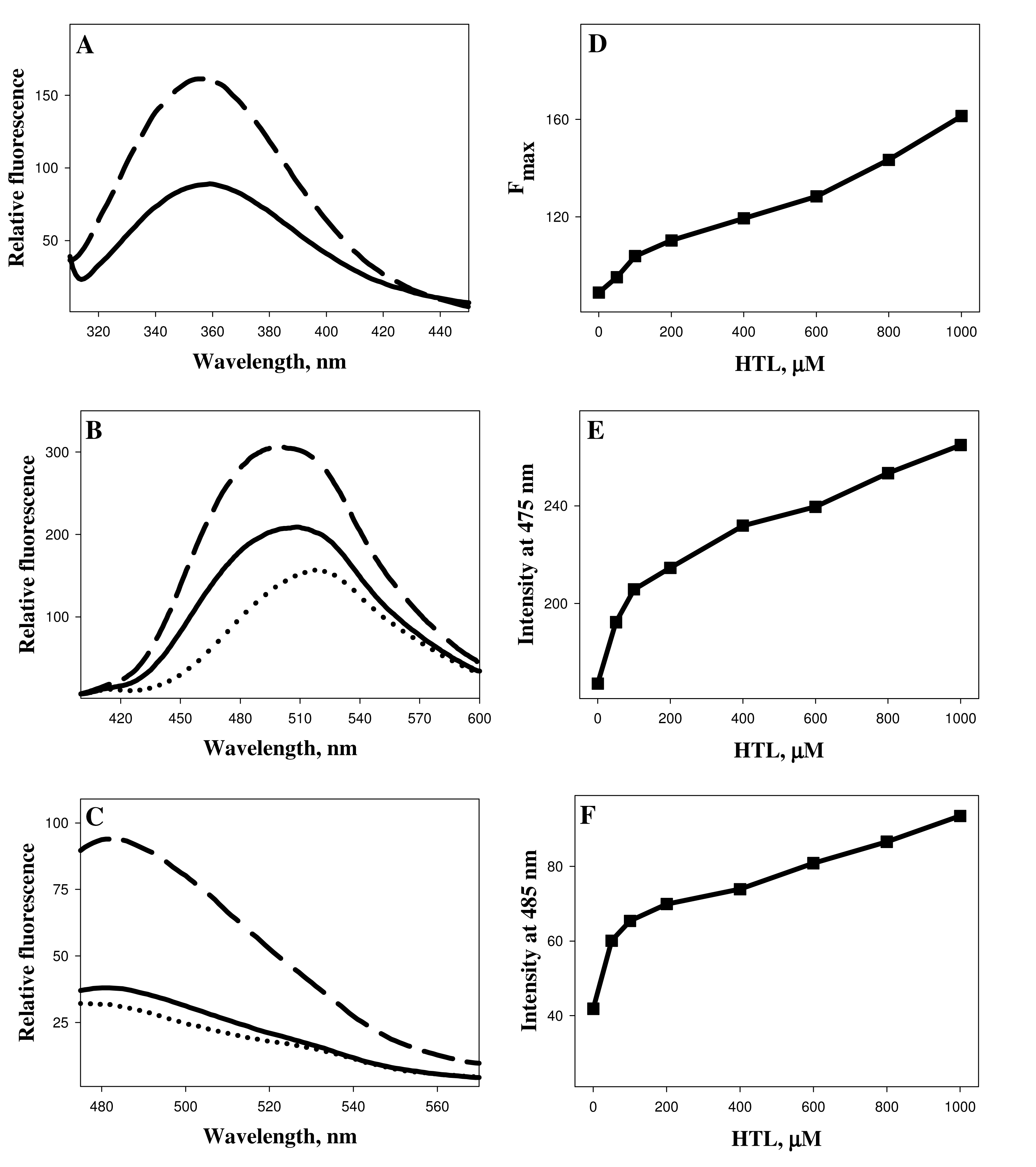

Supplement: S2 Fig — Fluorescence spectra of CN modified with varying concentrations of HTL. Left panel depicts intrinsic fluorescence (A), ANS binding assay (B) and ThT binding assay (C). Right panel depicts Fmax as a function of HTL concentration (D), and λmax as a function of HTL concentrations (E and F). To maintain clarity, only the representative curves of free dyes (dotted lines), control unmodified protein (solid lines) and protein modified with 1000 µM HTL (dashed lines) are shown. (TIF) [file pone.0116386.s002.tif]

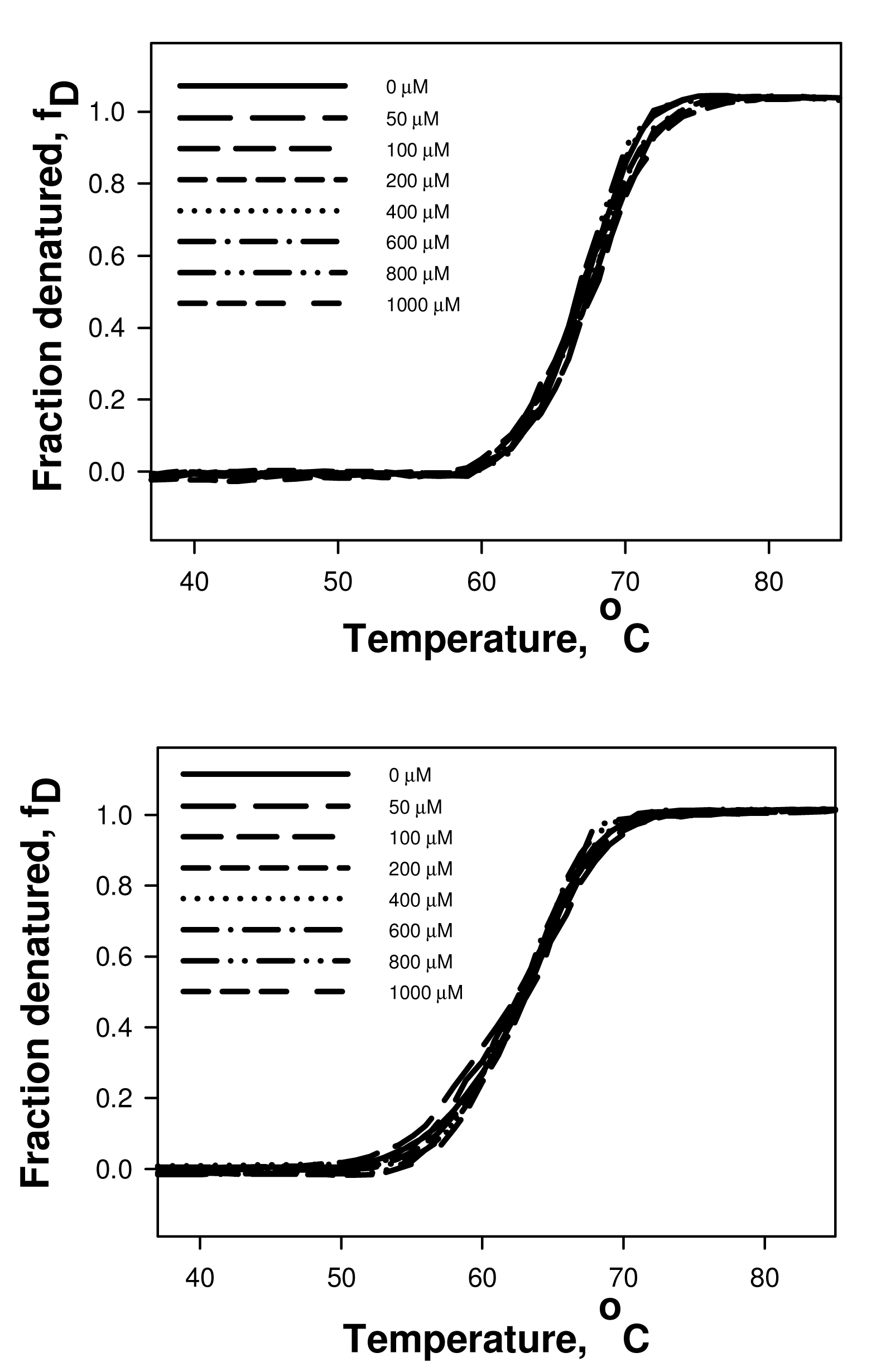

Supplement: S3 Fig — Representative thermal denaturation profiles of lysozyme and RNase-A. Denaturation curves of lysozyme (upper panel) and RNase-A (lower panel), in the absence and presence of various HTL concentrations at pH 7.4 phosphate buffer. (TIF) [file pone.0116386.s003.tif]

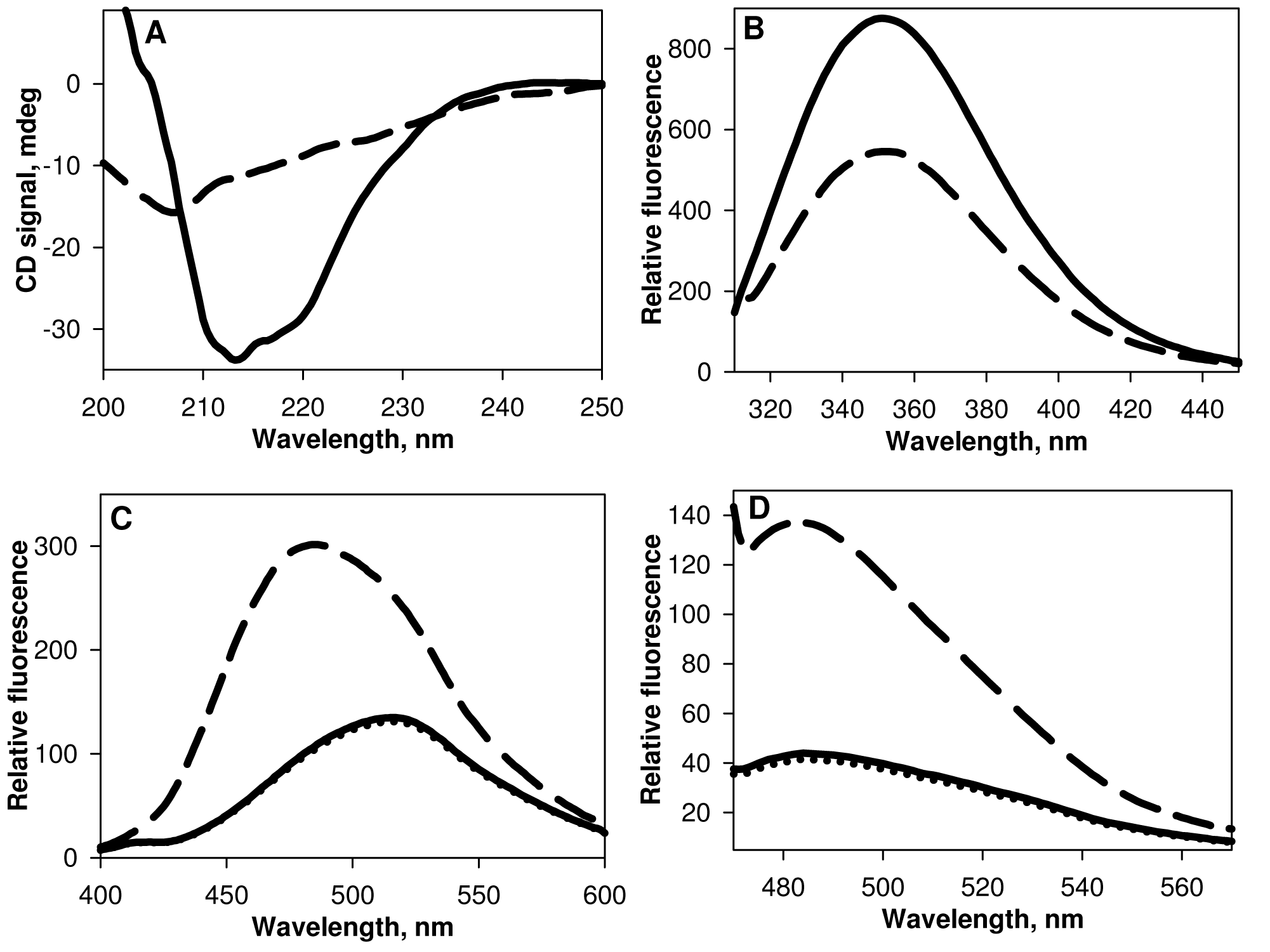

Supplement: S4 Fig — CD and fluorescence spectra of HTL-modified CA. Far UV-CD (A), intrinsic fluorescence spectra (B), ANS binding assay (C) and D-ThT binding assay (D) of HTL-modified CA. All spectra have been made in absence (solid lines) and in presence of 1000 µM HTL (dashed lines). In case of ANS and ThT binding assays, free dyes (dotted lines) have also been shown. (TIF) [file pone.0116386.s004.tif]

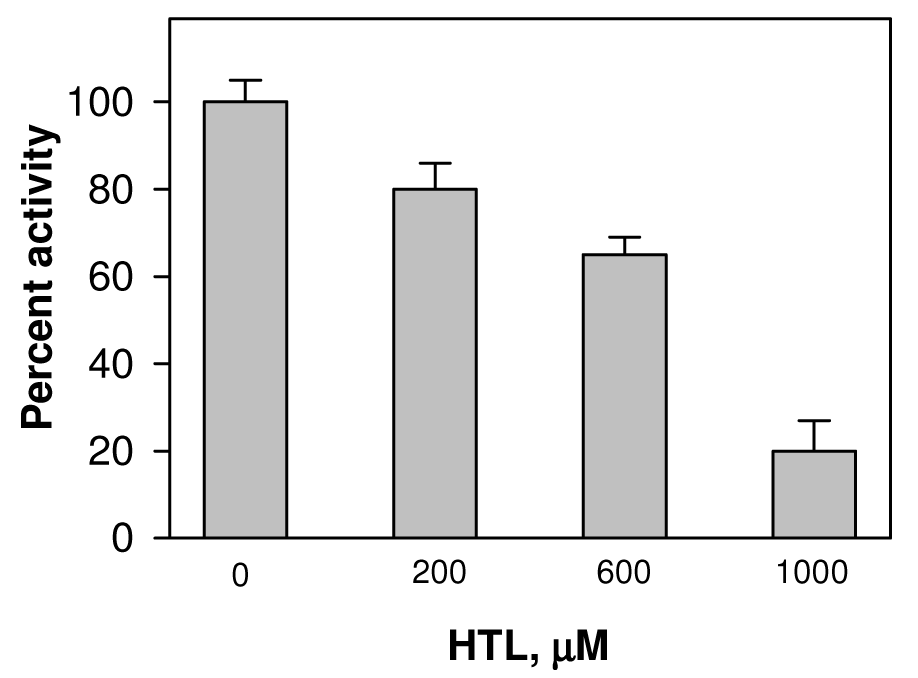

Supplement: S5 Fig — Effect of HTL-induced modification on CA enzymatic activity. Enzyme activity (in percent) was measured by monitoring the hydrolysis of p-nitrophenyl acetate (pNPA) at 400 nm. The enzyme concentration used was.03 mg ml−1 and substrate was kept 1 mM. (TIF) [file pone.0116386.s005.tif]
